# Effect of early and later prone positioning on outcomes in invasively ventilated COVID-19 patients with acute respiratory distress syndrome: analysis of the prospective COVID-19 critical care consortium cohort study

**DOI:** 10.1186/s13613-025-01422-6

**Published:** 2025-02-10

**Authors:** Andrew J. Simpkin, Bairbre A. McNicholas, David Hannon, Robert Bartlett, Davide Chiumello, Heidi J. Dalton, Kristen Gibbons, Nicole White, Laura Merson, Eddy Fan, Mauro Panigada, Giacomo Grasselli, Anna Motos, Antoni Torres, Ferran Barbé, Pauline Yeung Ng, Jonathon P. Fanning, Alistair Nichol, Jacky Y. Suen, Gianluigi Li Bassi, John F. Fraser, John G. Laffey, on behalf of the COVID-19 Critical Care Consortium

**Affiliations:** 1 School of Mathematical and Statistical Sciences, University of Galway, Galway, Ireland; 2 Department of Anaesthesia and Intensive Care Medicine, School of Medicine, Clinical Sciences Institute, Galway University Hospital, Saolta University Healthcare Group, Galway, H91 YR71 Ireland; 3 School of Medicine, College of Medicine, Nursing and Health Sciences, University of Galway, Galway, Ireland; 4 University of Michigan Medical Center, Ann Arbor, MI USA; 5 Ospedale San Paolo, Milan, Italy; 6 University of Milan, Milan, Italy; 7 INOVA Fairfax Medical Center, Heart and Vascular Institute, Falls Church, VA USA; 8 Child Health Research Centre, The University of Queensland, Brisbane, QLD Australia; 9 Children’s Intensive Care Research Program, Child Health Research Centre, The University of Queensland, Brisbane, QLD Australia; 10 ISARIC, Pandemic Sciences Institute, University of Oxford, Oxford, UK; 11 University of Toronto, Interdepartmental Division of Critical Care Medicine, Toronto, ON Canada; 12 Fondazione IRCCS Ca’ Granda, Ospedale Maggiore Policlinico Di Milano, Department of Anesthesia, Intensive Care and Emergency. Milano, Lombardia, Italy; 13 Centro de Investigación Biomedica En Red - Enfermedades Respiratorias (CIBERES), Madrid, Spain; 14 Institut d’Investigacions Biomediques August Pi I Sunyer (IDIBAPS), Barcelona, Universitat de Barcelona, Barcelona, Spain; 15 Servei de Pneumologia, Hospital Clinic, University of Barcelona, Barcelona, Spain; 16 Institució Catalana de Recerca I Estudis Avançats, Barcelona, Spain; 17 Translational Research in Respiratory Medicine, Respiratory Dept, Hospital Universitari Aranu de Vilanova and Santa Maria, Lleida, Spain; 18 Critical Care Medicine Unit, University of Hong Kong and Queen Mary Hospital, Hong Kong, China; 19 University College Dublin-Clinical Research Centre at St Vincent’s University Hospital, Dublin, Ireland; 20 Critical Care Research Group, The Prince Charles Hospital, Brisbane, Australia; 21 School of Pharmacy and Medical Sciences, Griffith University, Southport, Australia; 22 University of Queensland, Brisbane, Australia; 23 Uniting Care Hospitals, Brisbane, Australia; 24 Wesley Medical Research, Brisbane, Australia; 25 School of Public Health and Social Work, Faculty of Health, Queensland University of Technology, Brisbane, Australia; 26 Inserm, CHU Nantes, Center for Research in Transplantation and Translational 16 Immunology, UMR 1064, Nantes Université, F-44000 Nantes, France

**Keywords:** COVID-19, Cox proportional hazards models, Invasive mechanical ventilation, Prone positioning, Proning

## Abstract

**Background:**

Prone positioning of patients with COVID-19 undergoing invasive mechanical ventilation (IMV) is widely used, but evidence of efficacy remains sparse. The COVID-19 Critical Care Consortium has generated one of the largest global datasets on the management and outcomes of critically ill COVID-19 patients. This prospective cohort study investigated the association between prone positioning and mortality and in particular focussed on timing of treatment.

**Methods:**

We investigated the incidence, demographic profile, management and outcomes of proned patients undergoing IMV for COVID-19 in the study. We compared outcomes between patients prone positioned within 48 h of IMV to those (i) never proned, and (ii) proned only after 48 h.

**Results:**

3131 patients had data on prone positioning, 1482 (47%) were never proned, 1034 (33%) were proned within 48 h and 615 (20%) were proned only after 48 h of commencement of IMV. 28-day (hazard ratio 0.82, 95% confidence interval [CI] 0.68, 0.98, p = 0.03) and 90-day (hazard ratio 0.81, 95% CI 0.68, 0.96, p = 0.02) mortality risks were lower in those patients proned within 48 h of IMV compared to those never proned. However, there was no evidence for a statistically significant association between prone positioning after 48 h with 28-day (hazard ratio 0.93, 95% CI 0.75, 1.14, p = 0.47) or 90-day mortality (hazard ratio 0.95, 95% CI 0.78, 1.16, p = 0.59).

**Conclusions:**

Prone positioning is associated with improved outcomes in patients with COVID-19, but timing matters. We found no association between later proning and patient outcome.

**Supplementary Information:**

The online version contains supplementary material available at 10.1186/s13613-025-01422-6.

## Introduction

Prone positioning (PP) of patients with severe COVID-19, whether invasively mechanically ventilated or receiving advanced respiratory support (termed ‘awake’ proning), received considerable attention during the early phases of the COVID-19 pandemic. PP has previously been demonstrated to improve survival for non–COVID patients with moderate-to-severe acute respiratory distress syndrome (ARDS) receiving invasive mechanical ventilation (IMV) [1]. However, there are potentially important differences in the pathophysiology of COVID ARDS compared to ‘classic’ ARDS. The early stages of COVID-ARDS are characterised by a higher respiratory system compliance [2], with microangiopathy induced alveolar capillary microthrombi leading to higher deadspace fraction and higher V/Q mismatch [3] than in ‘classic’ ARDS. Given this, it is important not to extrapolate directly from studies of PP in ‘classic’ ARDS to COVID-19 ARDS.

The effect of prone position in invasively ventilated patients with severe COVID-19 remains unclear. A recent systematic review [4] found nine randomised controlled trials (RCT) and 23 observational studies reporting on the use of prone positioning for patients with COVID-19. The majority of these studies focused on proning of awake patients [5], and both the RCTs and observational studies found that awake proning significantly reduced the intubation rate among such patients. In terms of mortality, two meta-analyses of RCTs found no evidence for a benefit among the awake proned population, but there were no RCTs for patients undergoing IMV [4, 6].

The effect of the timing of PP may also be of importance. In ‘classic’ ARDS, the PROSEVA study found that early PP was effective, while there is no data demonstrating the efficacy of later or ‘rescue’ PP [1]. A meta-analysis of two observational studies of patients on IMV, found no evidence for an effect of proning on mortality [7, 8]. However, these studies compared patients proned at any time following commencement of IMV, i.e. both early and later proning, to those who were never proned. Camporota et al. reported patients with COVID-19 ARDS who responded to PP received earlier PP than non-responders, and early PP was associated with improved survival [9]. Conversely, Le Terrier et al. did not demonstrate any survival benefit of early PP (within 24h) compared to later proning in their cohort study [10]. Given this, it is important to determine separately the potential for early proning and for later proning to affect the outcome of COVID-19 induced ARDS.

Since January 2020, the COVID-19 Critical Care Consortium (CCCC), in collaboration with the International Severe Acute Respiratory and Emerging Infection Consortium (ISARIC) group have generated the largest global dataset on the demographics, management, and outcomes of critically ill COVID-19 patients. This dataset includes patients who were managed before and after the introduction of disease modifying therapies for COVID-19 including dexamethasone and tocilizumab in 2021 [11]. The CCCC currently includes 354 hospitals across 64 countries [12]. We aimed to examine the incidence, timing, and outcomes of prone positioning for patients with COVID-19 undergoing IMV. Our primary objective was to determine the effect of early (within 48 h of IMV commencement), and later proning (only after 48 h of IMV) compared to patients never proned. Our hypothesis was that the timing of proning is an important factor in its efficacy when treating COVID-19 patients undergoing IMV.

## Methods

### Study design and setting

The COVID-19 Critical Care Consortium (CCCC) registry is a global database enrolling COVID-19 patients requiring ICU care (Trial registration ACTRN12620000421932) [12]. Hospitals participating in CCCC obtained approval from their local institutional review board and received waivers of informed consent for all patients. A complete summary of recruiting sites, corresponding ethics/regulatory approvals, contributors, and collaborators is included in online materials. CCCC collaborates with the International Severe Acute Respiratory and Emerging Infection Consortium (ISARIC).

### Participants

Patients enrolled in the CCCC database from 1st January 2020 up to 14th February 2023 with laboratory-confirmed (real-time polymerase chain reaction) SARS-CoV-2 infection and upon first admission to ICU receiving invasive mechanical ventilation (IMV) for any cause were included. Those patients transferred from other ICUs already undergoing IMV were excluded. Patients missing final outcome, with unconfirmed ARDS (no bilateral infiltrates on x-ray) or P/F (PaO2/FiO2) over 300 were excluded.

### Study outcomes, data sources, measurements, and definitions

As described in the CCCC study protocol [12], data on demographics, comorbidities, clinical symptoms, and laboratory values were collected by clinical/research staff in all participating ICUs and recorded in an electronic case report form up to 28 days from commencement of IMV. Geographical regions were countries grouped into three categories: “Europe”, “rest of the world high income”, e.g., USA and Japan, and “rest of the world middle income”, e.g., Brazil (based on World Bank Gross National Income Classification). The primary outcome was all-cause mortality within 28 days of commencing IMV, with a secondary outcome of mortality within 90 days of commencing IMV.

### Prone position data definition

Our primary exposure was to assess whether proning within 48 h was beneficial, so we compared those who were proned early (within 48 h of IMV commencement) to those proned only after 48 h and to those never proned in a three-way comparison. We chose 48 h as the cut-off for the early proning group based on the PROSEVA study, which enrolled patients over a similar timeframe.

### Statistical analysis

To investigate the association of prone positioning with mortality we used a Cox proportional hazards model with death within 28 days of commencing IMV as the primary outcome, and repeated this using death within 90 days [13]. For the 28-day ICU mortality outcome, patients were censored if they were discharged from ICU before 28 days or were still alive in ICU on day 28. For the 90-day hospital mortality outcome, patients were censored if they were discharged from hospital before 90 days or were still alive in hospital on day 90. In each model, we adjusted for possible confounders based on previous research on prone positioning and mortality in ICU [6, 14, 15], the complete list of variables is available in online supplement. To account for missing data, we used multiple imputation via chained equations with 20 imputed datasets (full information on missing data is provided in the online supplement). Cox models performed and hazard ratios (HR) were combined using Rubin’s rules [16]. We also present a propensity score matched analyses for the binary exposure of never proned against (a) proned within 48 h and (b) proned only after 48 h, details of which are included in our online supplement. To investigate regional differences, we repeat our Cox regression analysis separately in European countries, non-European high-income countries, and non-European middle-income countries.

A secondary analysis focussed on the effect of prone positioning on PaO2 to FiO2 ratio (P/F) before, during and after prone positioning. Here a linear mixed model with random intercepts and slopes was employed to allow for correlation within and between patients. Time was measured via the day of P/F measurement and a linear spline was added with a knot placed at the day of first prone positioning. The effect of proning on P/F is captured through this linear spline, whose coefficient tells us about the change in P/F trend after proning. As such, this model can only be applied to those who were proned at some stage. All analyses were carried out in R v4.3 and in accordance with STROBE Guidelines [17].

## Results

**Fig. 1:**
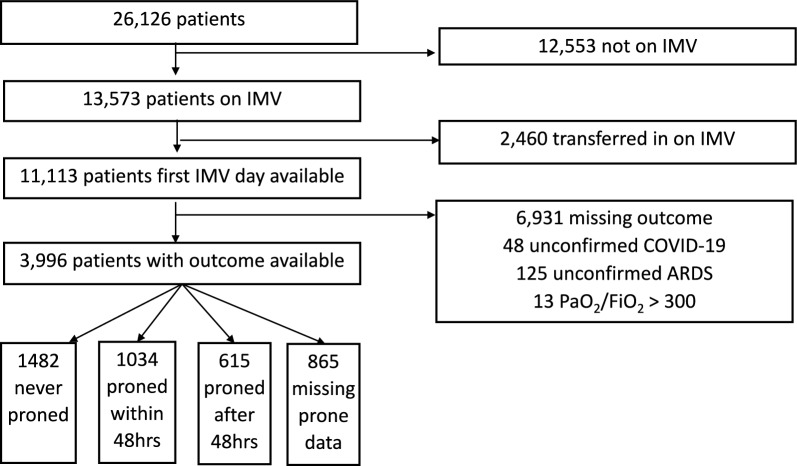
Flow chart for outcome analysis. IMV = invasive mechanical ventilation

A total of 3996 patients undergoing IMV were included in the cohort for final analysis (Fig. 1). Among these patients, 3131 patients had data on prone positioning, with 865 missing but included in our statistical analysis via multiple imputation. Of these, 1482 (47%) were never proned and 1649 (53%) were proned at some time, of whom 1034 (33%) were proned within 48 h of IMV commencement. Among those only proned after 48 h, the median and mean time of first proning were 4 and 5.5 days respectively, with standard deviation 5.7 days and interquartile range 3 days (Figure s1, s2). The P/F ratio on day of first proning was similar between those proned within and after 48 h (Figure s3).

### Demographics of PP use in invasively ventilated COVID-19 patients

We describe the characteristics of 3131 patients with data available on proning. Table 1 compares those who were proned within 48 h of IMV to those never proned and proned after 48 h of IMV. Patients proned within 48 h were more likely to be female, European, with lower prevalence of diabetes, hypertension and chronic cardiac disease than patients who were never proned or proned only after 48 h. Compared to those never proned, patients who were proned within 48 h had higher average PEEP, but lower average S/F (SaO2/FiO2) and P/F ratios, were more likely to receive heparin and corticosteroids, and spent longer undergoing IMV and in ICU on average. Those proned within 48 h were less likely to suffer complications such as heart failure. Among those patients who died, time to death was earlier on average in those never proned compared to those proned within 48 h.

**Table 1:** Characteristics, illness severity profile, and outcomes of ARDS patients with COVID-19 on mechanical ventilation stratified by proning group

	n	Never proned^1^	Proned only after 48 hours^1^	Proned within 48 hours^1^
		1482 (47%)	615 (20%)	1034 (33%)
Age (years)	3131	58 (16)	58 (14)	57 (13)
Sex (male)	3129	553 (37%)	195 (32%)	301 (29%)
Body Mass Index (kg/m^2^)	2855	29 (8)	30 (7)	31 (8)
Region	3111			
Europe		219 (15%)	170 (28%)	345 (34%)
Rest of World middle income		975 (66%)	391 (64%)	615 (60%)
Rest of World High income		282 (19%)	52 (8.5%)	62 (6.1%)
Comorbidities				
Diabetes	2993	502 (36%)	188 (32%)	284 (28%)
Hypertension	3105	736 (50%)	311 (51%)	474 (46%)
Malignant neoplasm	2961	67 (4.7%)	23 (4.0%)	30 (3.1%)
Chronic cardiac disease	3066	307 (21%)	104 (17%)	116 (11%)
Organ Injury/Ventilation Parameters				
SaO_2_/FiO_2_ ratio	2397	125 (54)	116 (43)	113 (42)
PaO_2_/FiO_2_ ratio	2538	110 (57)	100 (50)	95 (50)
Positive end-expiratory pressure (PEEP, cmH_2_O)	2233	11.1 (3.6)	12.1 (3.4)	12.5 (3.4)
Need for extracorporeal membrane oxygenation (ECMO)	2618	107 (8.5%)	33 (6.6%)	99 (12%)
Lowest Tidal Volume (mL)	1748	402 (122)	401 (121)	401 (121)
Highest respiratory rate (breaths per minute)	2239	26 (9)	26 (8)	26 (7)
Medications				
Heparin	1186	542 (84%)	198 (91%)	303 (95%)
Corticosteroids	2838	942 (69%)	474 (81%)	729 (82%)
Antibiotics	2693	1254 (95%)	529 (95%)	780 (95%)
Complications				
Heart failure	2525	87 (7.0%)	20 (3.9%)	21 (2.7%)
Stroke	2670	52 (4.0%)	20 (3.6%)	23 (2.8%)
Outcomes				
Days on IMV	3047	14 (18)	20 (19)	20 (23)
Days in ICU	2981	18 (18)	25 (23)	25 (25)
Days in hospital	2892	28 (33)	34 (38)	35 (36)
Days from hospital admission to IMV	2981	4.8 (7.6)	4.4 (5.2)	4.1 (7.0)
Days from ICU admission to IMV	2858	1.88 (5.14)	2.53 (4.50)	1.65 (3.58)
28-day ICU mortality	3131	693 (47%)	285 (46%)	391 (38%)
90-day hospital mortality	3131	786 (53%)	341 (55%)	472 (46%)
Days to death	1622	14 (24)	17 (16)	21 (35)
Cause of death	1551			
Cardiac failure		53 (7.0%)	16 (4.8%)	26 (5.6%)
Cerebrovascular accident		6 (0.8%)	6 (1.8%)	10 (2.2%)
Haemorrhagic shock		12 (1.6%)	4 (1.2%)	5 (1.1%)
Multi-organ failure		257 (34%)	137 (41%)	187 (40%)
Other		52 (6.9%)	12 (3.6%)	18 (3.9%)
Respiratory failure		270 (36%)	134 (40%)	184 (40%)
Septic shock		100 (13%)	22 (6.6%)	35 (7.5%)

1. n (%); Mean (SD)

### Patient outcomes

**Fig. 2:**
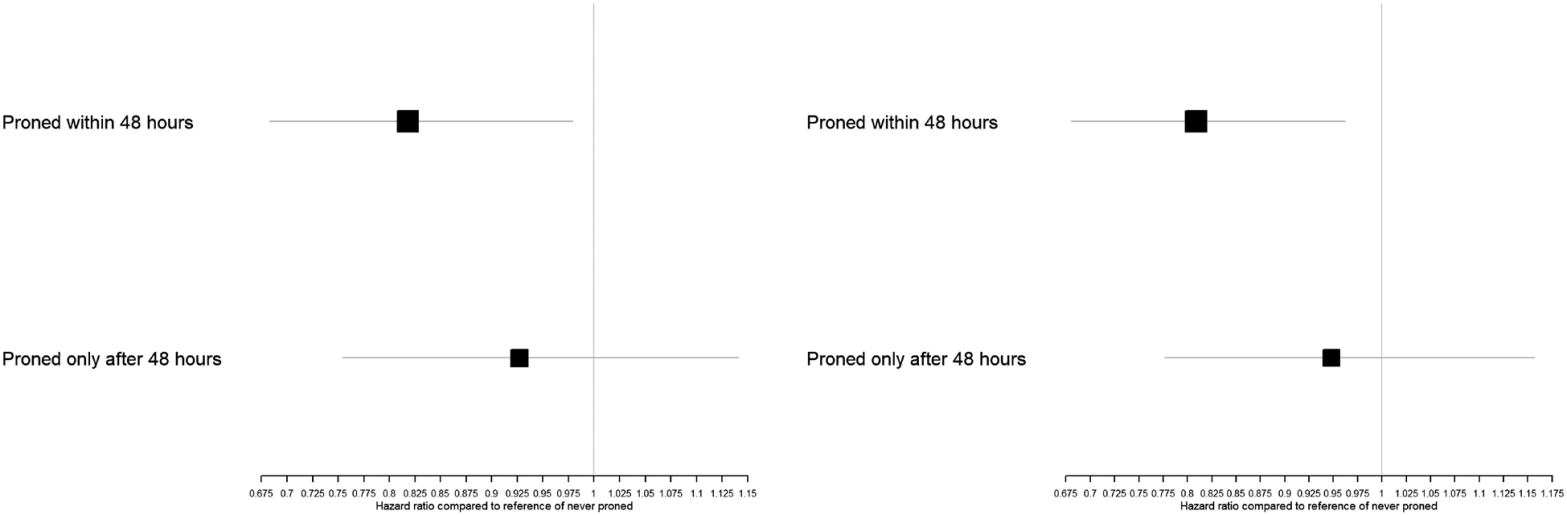
Hazard ratios for proning within and only after 48 h compared to never proning for outcomes of 28-day ICU mortality (left) and 90-day hospital mortality (right)

In a three-category comparison, we found a beneficial association of proning within 48 h of IMV on 28-day (HR 0.82, 95% confidence interval [CI] 0.68, 0.98, p = 0.029) and 90-day (HR 0.81, 95% CI 0.68, 0.96, p = 0.017) mortality compared to never proning. Those proned only after 48 h had no evidence for a reduced risk of 28-day (HR 0.93, 95% CI 0.75, 1.14, p = 0.469) and 90-day (HR 0.95, 95% CI 0.78, 1.16, p = 0.590) mortality compared to those never proned (Table 2, Fig. 2).

**Table 2:** Hazard ratios for the association between prone positioning and survival, using two outcomes (28-day ICU and 90-day hospital mortality) against a three-level proning exposure: never proned, proned within 48 h, proned only after 48 h

Outcome	Prone group	Hazard Ratio	Lower 95%	Upper 95%	*p*-value
*28-day ICU mortality*	*Never versus Later versus Early*				
	Never Proned (Reference)	1			
	Proned within 48 h	0.818	0.683	0.979	0.029
	Proned only after 48 h	0.927	0.754	1.141	0.469
*90-day hospital mortality*	*Never versus Later versus Early*				
	Never Proned (Reference)	1			
	Proned within 48 h	0.809	0.680	0.962	0.017
	Proned only after 48 h	0.948	0.776	1.157	0.590

Each Cox regression model is adjusted for the same set of covariates

**Fig. 3:**
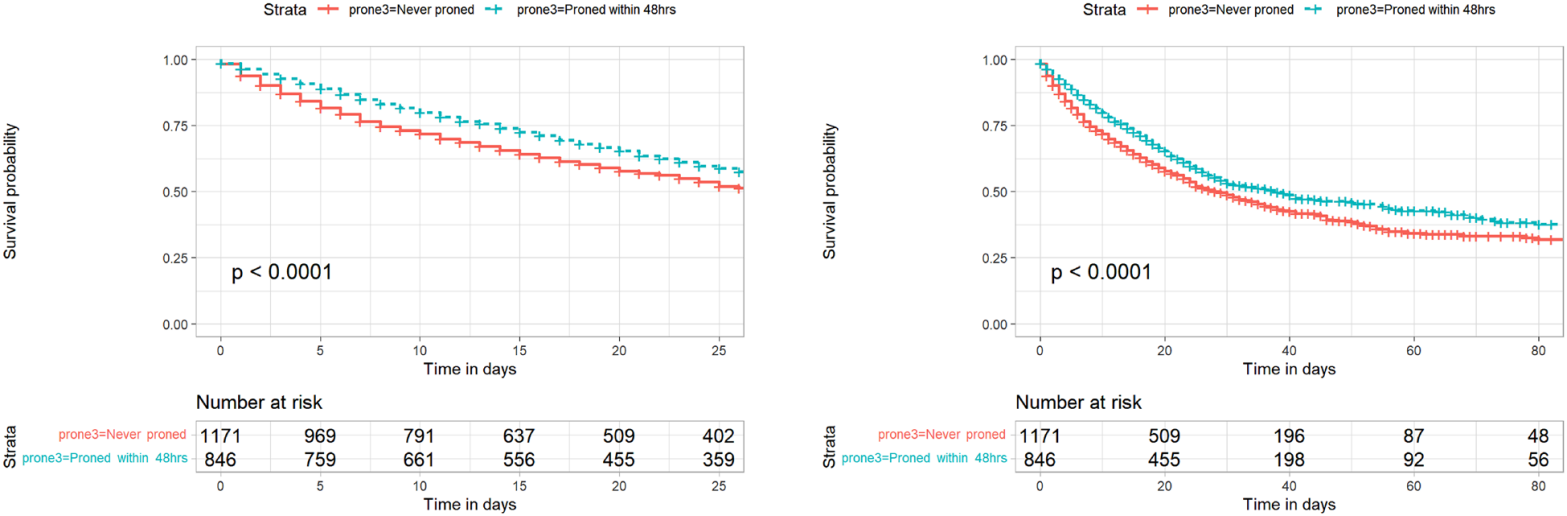
Kaplan Meier plots of propensity matched cohorts showing 28-day ICU survival (left) and 90-day hospital survival (right) comparing those proned within 48 h against a matched cohort of those never proned

In our propensity score matched analyses, patients who were proned within 48 h had reduced risk of 28-day (HR 0.85, 95% confidence interval [CI] 0.73, 1.00, p = 0.045) and 90-day mortality (HR 0.85, 95% CI 0.74, 0.99, p = 0.030) mortality compared to matched patients who did not receive early proning (Table 3; Fig. 3).

**Table 3:** Hazard ratios for the association between prone positioning and survival, using two outcomes (28-day and 90-day mortality) in a ***propensity score matched*** cohorts (never proned vs. proned within 48 h, and never proned vs. proned only after 48 h)

Outcome	Prone group	Hazard Ratio	Lower 95%	Upper 95%	*p*-value
*28-day ICU mortality*	*Never versus Later versus Early*				
	Never Proned (Reference)	1			
	Proned within 48 h	0.853	0.730	0.997	0.045
	Proned only after 48 h	1.002	0.852	1.178	0.984
*90-day hospital mortality*	*Never versus Later versus Early*				
	Never Proned (Reference)	1			
	Proned within 48 h	0.854	0.741	0.985	0.030
	Proned only after 48 h	1.022	0.880	1.188	0.773

Each patient who was proned was matched with two individuals never proned

In a stratified analysis by region (Table 4; Figures s4-s6), the main findings only held in those high-income non-European countries where proning within 48 h was associated with a reduced risk of 28-day (HR 0.80, 95% CI 0.67, 0.96, p = 0.015) and 90-day (HR 0.82, 95% CI 0.70, 0.96, p = 0.015) mortality compared to no proning.

**Table 4:** Hazard ratios for the association between prone positioning and survival, using two outcomes (28-day ICU and 90-day hospital mortality) against a three-level proning exposure: never proned, proned within 48 h, proned only after 48 h

Outcome	Prone group	Hazard Ratio	Lower 95%	Upper 95%	*p*-value
Europe (n = 902)					
*28-day ICU mortality*	*Never versus Later versus Early*				
	Never Proned (Reference)	1			
	Proned within 48 h	1.012	0.700	1.465	0.948
	Proned only after 48 h	1.112	0.721	1.715	0.626
*90-day hospital mortality*	*Never versus Later versus Early*				
	Never Proned (Reference)	1			
	Proned within 48 h	0.944	0.661	1.349	0.750
	Proned only after 48 h	1.119	0.745	1.681	0.582
High-income rest of the world (n = 2387)					
*28-day ICU mortality*	*Never versus Later versus Early*				
	Never Proned (Reference)	1			
	Proned within 48 h	0.803	0.672	0.959	0.015
	Proned only after 48 h	0.956	0.788	1.159	0.643
*90-day hospital mortality*	*Never versus Later versus Early*				
	Never Proned (Reference)	1			
	Proned within 48 h	0.818	0.697	0.961	0.015
	Proned only after 48 h	0.985	0.821	1.182	0.870
Middle-income rest of the world (n = 412)					
*28-day ICU mortality*	*Never versus Later versus Early*				
	Never Proned (Reference)	1			
	Proned within 48 h	0.968	0.673	1.394	0.862
	Proned only after 48 h	1.081	0.753	1.553	0.672
*90-day hospital mortality*	*Never versus Later versus Early*				
	Never Proned (Reference)	1			
	Proned within 48 h	0.968	0.673	1.393	0.860
	Proned only after 48 h	1.136	0.795	1.622	0.482

Each Cox regression model is adjusted for the same set of covariates. Models were repeated by geographical region (Europe [n = 916]], high-income rest of the world [n = 2393], and middle-income rest of the world [n = 412])

**Fig. 4:**
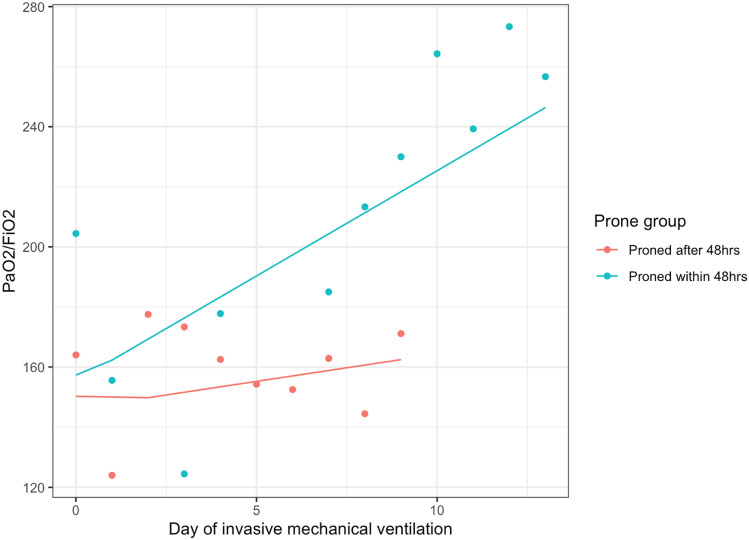
Fitted and actual PaO2/FiO2 ratios for two patients using a linear mixed model with a linear spline term allowing a change in slope on the day of first proning

In our analysis of changes in P/F ratio over time during IMV (Table 5, Fig. 4), we found no significant change in P/F ratio before proning (0.26 change per day, 95% CI − 0.60 to 1.13 per day, p = 0.551). However, upon proning this slope change by 2.04 units per day (95% CI 1.14, 2.95, p < 0.001) suggesting that P/F ratio changed by 2.3 units per day (i.e. 2.04 + 0.26) after proning.

**Table 5:** Longitudinal model of PaO2/FiO2 ratio during invasive mechanical ventilation among those proned at any stage. Results show the change in P/F before and after prone positioning

Predictors	Estimates	Lower 95%	Upper 95%	*p*-value
PaO2/FiO2 on day 0 of IMV	116.38	113.32	119.44	< 0.001
Slope of (i.e. change in) PaO2/FiO2 before prone positioning	0.26	− 0.60	1.13	0.551
Change to slope after first prone position	2.04	1.14	2.95	< 0.001

## Discussion

In this study, we investigated the patterns of use, and the potential for early and later prone positioning (PP) to impact on outcomes, in patients with COVID-19 induced severe respiratory failure receiving IMV. Our first major finding is that the use of proning was substantially higher than expected, with 53% of patients proned while receiving IMV. We also uncovered discordant results for the efficacy of proning, in that the potential for benefit depended on the timing of a first proning attempt. While proning within 48 h of commencement of IMV was associated with improved outcomes, there was no demonstrable benefit for later proning commencement over usual care. The upward trajectory of improvement in P/F ratio over time was quicker and steeper after prone positioning, as demonstrated via a model of longitudinal P/F ratio during IMV. Here we showed that proning was associated with a greater rate of improvement in P/F over time. Among those proned in the first 48 h 4.7% were co-infected, while in those never proned 5.5% were co-infected (p = 0.8).

### Current knowledge regarding PP in COVID-19 patients requiring IMV

PP of patients undergoing IMV has been used frequently in patients with COVID-19. The evidence base for prone positioning of patients with moderate-severe ‘classic’ ARDS is clear [1]. In addition, the potential for awake prone positioning to reduce the need for IMV [6, 15], and to reduce mortality [5, 18], has been demonstrated in COVID-19 patients receiving advance respiratory support. However, the distinct differences between COVID-19 ARDS and ‘classic’ ARDS, in terms of lung pathophysiology and treatment responsiveness, and the difference between COVID-19 patients receiving advanced respiratory support versus IMV, raises caution regarding simple extrapolation of therapeutic strategies across these conditions.

The evidence base for PP of invasively ventilated patients with COVID-19 ARDS is surprisingly sparse. Several studies have demonstrated improvements in lung aeration and recruitment and systemic oxygenation [19, 20], reduced shunt [21], and improved haemodynamics [22] with the use of PP in invasively ventilated COVID-19 patients, but a consistent effect on mortality is not demonstrated. Le Terrier et al., in a propensity matched analysis, did not find a benefit of early (within 24Hrs) versus no early PP (i.e. both later PP and never proned patients) on clinical outcomes in invasively ventilated COVID-19 patients [10]. In this analysis, of the 1504 patients that did not receive early PP, 1013 were proned with a median delay of 3 days. The outcomes in the ‘never’ versus ‘late’ proned patient groups are not distinguished in their analysis, and all their patients were European. Stilma et al., in their analysis of 734 invasively ventilated COVID-19 patients across 22 ICUs in the Netherlands found no association between PP and outcome [8]. Langer et al. reported that patients receiving IMV in the first wave of the pandemic in Italy had more severe disease and a higher mortality rate, while patients that responded to PP did not seem to have improved outcomes [23]. In contrast, Mathews et al. in the US, in an inverse probability treatment weighting analysis, reported that in-hospital mortality was lower in mechanically ventilated hypoxemic patients with COVID-19 treated with early proning (within first 48Hrs) [24].

A systematic review and meta-analysis of seven retrospective cohort studies [25] that enrolled 5216 patients reported that ICU mortality risk was twice as high in the prone group, while there was no difference in hospital or overall mortality. A key issue in these analyses is that no distinction was made in regard to the timing of the proning, with all proned (i.e. early and later ‘rescue’ proning) included in the treatment groups.

The timing of commencement of PP is important because of the potential for PP to homogenise lung stress and thereby reduce the potential for additional ventilator induced lung injury. In ‘classic’ ARDS the early use of PP is given a strong recommendation in the recently published ESICM guidelines for ARDS management [26]. This guideline also recommends early proning for COVID-19 ARDS, but acknowledges that there is no RCT evidence to support this recommendation [26].

The need for a clear determination of the effect of PP in this population is underlined by a number of findings. First, studies have demonstrated that implementation of PP in patients with moderate-severe COVID-19 ARDS requiring IMV is associated with higher resource use [27], particularly in terms of the requirement for healthcare personnel to prone patients safely. Second, there is an increased frequency of complications, including haemodynamic instability and pressure ulcers in patients that undergo PP [28]. Third, the use of PP appears to be declining after an initial high uptake in the earlier phases of the COVID-19 pandemic [29]. Consequently, there is an urgent need to determine both the effectiveness and optimal timing of PP in this population.

### Clinical implications

Our study addresses limitations in the existing literature, and provides significant new insights into the effects of PP, and particularly the importance of timing of PP in patients with COVID-19 managed with IMV. Firstly, we demonstrate that early PP is associated with improved outcomes. Patients who were proned early had lower risk of 28-day ICU mortality and 90-day hospital mortality compared to those who were never proned. Among those patients who died, time to death was earlier on average in those never proned compared to those proned early. A propensity score matched analysis confirmed these findings of a reduced 28-day and 90-day hospital mortality risk in patients receiving early PP. Those proned early were also less likely to suffer complications such as heart failure.

Of equal importance, we could find no evidence that later proning was associated with improved outcomes in this population. Those proned after 48 h had no reduction in the risk of 28-day or 90-day mortality compared to those never proned. It is clear from these findings that, for proning to be effective, it should be instituted within 48 h of commencement of IMV.

### Strengths and limitations

A strength of this study is its size and diversity, with 3996 patients from 35 countries included. However, since this was an observational study, we cannot draw any causal conclusions about proning–that may require future randomised studies in this population. While the possibility of immortal time bias related to the timing of proning exists, this is less likely to be important given our focus on early proning within 48 h of IMV rather than at later time points. Furthermore, we compare different, time-independent prone position hypotheses.

The ability to prone patients early following IMV may reflect issues such as greater centre experience with the manoeuvre, staffing within the ICU etc., all of which may affect mortality by other mechanisms. In addition, we cannot exclude the possibility that that some patients undergoing later proning were deteriorating clinically, and underwent ‘rescue’ proning in an attempt to reduce a high risk of death. The finding that P/F ratio on day of first proning was similar between those proned within and after 48 h does suggest that this was not a major factor. Those proned early tended to have protective factors against mortality, e.g. less diabetes and hypertension. While we controlled for the observed confounding of observed data, there still may be unmeasured confounding by other, unmeasured protective factors linked to early prone positioning.

Potential between-hospital heterogeneity exists in admission criteria for ICU, indication for IMV, and use of adjuncts such as neuromuscular blockade. These were not standardised across countries and could have depended on local practices. We provide a stratified analysis by region, and this suggests our results were driven by those non-European high-income countries. It is possible that geographic differences in duration of proning sessions might explain these findings. Unfortunately, data on prone duration were not consistently available in this dataset. Our findings are consistent with prior studies [30] that identified differences between European and the non-European countries regarding ARDS severity profiles, and in the duration of invasive ventilation and length of stay in ICU across these regions. We suggest that this finding deserves further exploration in separate studies.

The database had high levels of missing data for some respiratory variables such as plateau pressure data, and data on plateau pressure, driving pressure and respiratory system compliance. However, our analysis included other respiratory dysfunction severity indices, in particular P/F ratio, which is the criterion used in the pivotal PROSEVA study by Guerin et al. as a criterion for prone positioning in the study population. Data on bacterial infection rates in the dataset also had a high rate of missing values. However, among those with data, there were no statistically significant differences between the prone groups. No data on life limiting measures were available in this study, and so we could not explore whether rates differed between the proning groups. We used multiple imputation to address missing data, with no significant differences in main exposures and outcomes between those complete case patients and patients with missing data (Table s1). Furthermore, our results were robust in a sensitivity analysis restricted to complete case patients (Table s2).

Another limitation of our study is the difference in timing from admission to MV, with those early proned having a shorter average time (4.1 days) to those never (4.8 days) or later proned (4.4 days). This is explained in part by the fact that the time from ICU admission to commencement of invasive MV is shorter in the group proned within 48 h. The changing nature of COVID-19 treatment from January 2020 to March 2023 may have influenced findings. For instance, early in the pandemic, some ICUs struggled to manage critically ill patients in understaffed and overwhelmed departments.

## Conclusions

Proning is a commonly used treatment in COVID-19 patients receiving IMV, and proning patients within 48 h of IMV initiation is associated with improved survival. Timing of proning is critical, as there was no significant effect of proning administered after 48 h.

## Supplementary Information

Additional file 1

## Data Availability

The datasets used and/or analysed during the current study are available from the corresponding author on reasonable request.

## Abbreviations

ARDS: Acute respiratory distress syndrome
CCCC: COVID-19 Critical Care Consortium
CI: Confidence interval
HR: Hazard ratio
ICU: Intensive care unit
IMV: Invasive mechanical ventilation
ISARIC: International Severe Acute Respiratory and Emerging Infection Consortium
PP: Prone positioning
RCT: Randomised controlled trial
