# Correction: Effect of early and later prone positioning on outcomes in invasively ventilated COVID-19 patients with acute respiratory distress syndrome: analysis of the prospective COVID-19 critical care consortium cohort study

**DOI:** 10.1186/s13613-025-01466-8

**Published:** 2025-03-28

**Authors:** Andrew J. Simpkin, Bairbre A. McNicholas, David Hannon, Robert Bartlett, Davide Chiumello, Heidi J. Dalton, Kristen Gibbons, Nicole White, Laura Merson, Eddy Fan, Mauro Panigada, Giacomo Grasselli, Anna Motos, Antoni Torres, Ferran Barbé, Pauline Yeung Ng, Jonathon P. Fanning, Alistair Nichol, Jacky Y. Suen, Gianluigi Li Bassi, John F. Fraser, John G. Laffey, on behalf of the COVID-19 Critical Care Consortium

**Affiliations:** 1 School of Mathematical and Statistical Sciences, University of Galway, Galway, Ireland; 2 Department of Anaesthesia and Intensive Care Medicine, School of Medicine, Clinical Sciences Institute, Galway University Hospital, Saolta University Healthcare Group, Galway, H91 YR71 Ireland; 3 School of Medicine, College of Medicine, Nursing and Health Sciences, University of Galway, Galway, Ireland; 4 University of Michigan Medical Center, Ann Arbor, MI USA; 5 Ospedale San Paolo, Milan, Italy; 6 University of Milan, Milan, Italy; 7 INOVA Fairfax Medical Center, Heart and Vascular Institute, Falls Church, VA USA; 8 Child Health Research Centre, The University of Queensland, Brisbane, QLD Australia; 9 Children’s Intensive Care Research Program, Child Health Research Centre, The University of Queensland, Brisbane, QLD Australia; 10 ISARIC, Pandemic Sciences Institute, University of Oxford, Oxford, UK; 11 University of Toronto, Interdepartmental Division of Critical Care Medicine, Toronto, ON Canada; 12 Fondazione IRCCS Ca’ Granda, Ospedale Maggiore Policlinico Di Milano, Department of Anesthesia, Intensive Care and Emergency. Milano, Lombardia, Italy; 13 Centro de Investigación Biomedica En Red - Enfermedades Respiratorias (CIBERES), Madrid, Spain; 14 Institut d’Investigacions Biomediques August Pi I Sunyer (IDIBAPS), Barcelona, Universitat de Barcelona, Barcelona, Spain; 15 Servei de Pneumologia, Hospital Clinic, University of Barcelona, Barcelona, Spain; 16 Institució Catalana de Recerca I Estudis Avançats, Barcelona, Spain; 17 Translational Research in Respiratory Medicine, Respiratory Dept, Hospital Universitari Aranu de Vilanova and Santa Maria, Lleida, Spain; 18 Critical Care Medicine Unit, University of Hong Kong and Queen Mary Hospital, Hong Kong, China; 19 University College Dublin-Clinical Research Centre at St Vincent’s University Hospital, Dublin, Ireland; 20 Critical Care Research Group, The Prince Charles Hospital, Brisbane, Australia; 21 School of Pharmacy and Medical Sciences, Griffith University, Southport, Australia; 22 University of Queensland, Brisbane, Australia; 23 Uniting Care Hospitals, Brisbane, Australia; 24 Wesley Medical Research, Brisbane, Australia; 25 School of Public Health and Social Work, Faculty of Health, Queensland University of Technology, Brisbane, Australia; 26 Inserm, CHU Nantes, Center for Research in Transplantation and Translational 16 Immunology, UMR 1064, Nantes Université, F-44000 Nantes, France

The Supplementary [CCCC consortium] was missing from this article and has now been uploaded.

The original article has been corrected.

## Electronic supplementary material

Below is the link to the electronic supplementary material.

Supplementary Material 1

